# Effects of shrub encroachment on soil organic carbon in global grasslands

**DOI:** 10.1038/srep28974

**Published:** 2016-07-08

**Authors:** He Li, Haihua Shen, Leiyi Chen, Taoyu Liu, Huifeng Hu, Xia Zhao, Luhong Zhou, Pujin Zhang, Jingyun Fang

**Affiliations:** 1 State Key Laboratory of Vegetation and Environmental Change, Institute of Botany, Chinese Academy of Sciences, Beijing, 100093, China; 2 University of the Chinese Academy of Sciences, Beijing, 100049, China; 3 Inner Mongolia Prataculture Research Center, Chinese Academy of Sciences, Hohhot, 010031, China; 4 Department of Ecology, College of Urban and Environment, and Key Laboratory of Earth Surface Processes of the Ministry of Education, Peking University, Beijing, 100871, China

## Abstract

This study aimed to evaluate the effect of shrub encroachment on soil organic carbon (SOC) content at broad scales and its controls. We conducted a meta-analysis using paired control data of shrub-encroached grassland (SEG) vs. non-SEG collected from 142 studies worldwide. SOC contents (0–50 cm) were altered by shrub encroachment, with changes ranging from −50% to + 300%, with an effect size of 0.15 (*p* < 0.01). The SOC contents increased in semi-arid and humid regions, and showed a greater rate of increase in grassland encroached by leguminous shrubs than by non-legumes. The SOC content decreased in silty and clay soils but increased in sand, sandy loam and sandy clay loam. The SOC content increment was significantly positively correlated with precipitation and temperature as well as with soil bulk density but significantly negatively correlated with soil total nitrogen. We conclude the main effects of shrub encroachment would be to increase topsoil organic carbon content. As structural equation model revealed, soils properties seem to be the primary factors responsible for the extent of the changes, coarse textured soils having a greater capacity than fine textured soils to increase the SOC content. This increased effect appears to be secondarily enhanced by climate and plant elements.

Over the last century, shrub encroachment, i.e., the increase in abundance and dominance of adjacent or local shrubs, has been observed in grasslands worldwide^1^,^2^,^3^. Shrub encroachment as a naturally occurring land cover transformation often accompanies the degradation of grasslands^4^,^5^. The shift from grasslands to shrub-encroached grasslands (SEG), which is often irreversible, can result in various ecological consequences, such as changes to biodiversity, soil organic carbon (SOC), and the regional carbon balance^6^,^7^,^8^.

Studies have found that shrub encroachment in grassland often changes the SOC content, but these studies have many uncertainties and have resulted in contradictory conclusions^9^,^10^. Some studies found that shrub encroachment increases SOC^11^,^12^,^13^. In contrast, shrub encroachment resulted in SOC decreases in some areas^7^,^14^,^15^ or caused negligible changes in others^16^,^17^. These contradictory results revealed a large regional variation in the impact of shrub encroachment on SOC. Recently, two meta-analyses of American and global datasets found that shrub encroachment would increase the SOC content at a global scale^8^,^18^. However, the geographical pattern of the SOC changes and their controlling factors remain unclear. Further exploration of the controlling factors affecting SOC changes in relation to shrub encroachment will help us understand the encroachment process and its ecological impact on the carbon cycle^10^,^19^.

The process of shrub encroachment is affected directly or indirectly by climate, soil conditions and local shrub types^3^,^20^. These factors can also influence the effect on SOC content induced by shrub encroachment. Jackson *et al*.^7^ identified a negative relationship between precipitation and changes in SOC and nitrogen content (STN) when grasslands were invaded by woody vegetation, with drier sites gaining and wetter sites losing SOC. A meta-analysis by Barge *et al*.^18^ found that the relationship between precipitation and SOC increment in response to shrub encroachment was generally negative^18^, however, the modeling result showed an opposite relationship^21^. These inconsistent findings suggest the need for further assessments.

Soil properties are perhaps one of the most important factors leading to the uncertainties observed previously^22^,^23^. Different components of SOC combined with differences in water content, pH and particle-size structure can contribute to the differences in the changes to SOC under shrub encroachment^24^. Soil texture interactions with other physical and chemical properties will affect SOC accumulation^25^. Thus, the soil texture determines the initial grassland SOC content, which also determines the potential SOC content following shrub invasion. Wheeler *et al*.^55^ found that in sandy loam soil, the accumulation of SOC as a result of shrub encroachment was approximately 23% higher than in clay loam soil with the same precipitation and temperature. Moreover, different “invasive” shrub species have different impacts on soils; most grasslands have been encroached by leguminous plants^10^, and their impacts on SOC compared with non-legumes are still unclear.

Moreover, the different approaches and scales used-e.g., some studies compared shrub patches with grass patches in SEG, whereas others compared SEG and non-SEG, treating SEG as a whole—make it difficult to draw general conclusions^20^,^26^. Fortunately, increasing worldwide data now provide an opportunity to further evaluate the patterns of SOC change following shrub encroachment and their underlying mechanisms.

We conducted a meta-analysis to explore the impact of shrub encroachment on SOC in relation to climate factors, soil properties, and shrub types. A total of 142 datasets from 41 publications across the world were collected. Using these data, we addressed the following two questions: (1) Does shrub encroachment increase the SOC content, and what is the geographical pattern of the SOC change? (2) How do climate, soil conditions and shrub types influence encroachment-induced SOC changes?

## Materials and Methods

### Data Sources

Data were mainly assembled from previous meta-analysis, literature reviews, or published data and so on. Keywords for searching in Web of Science and Google Scholar related literature included (1) shrub* OR bush* OR vegetation* OR woody* OR grassland* OR arid* OR sub-arid* OR savanna* OR steppe* OR shrubland* OR prairie* OR encroachment* OR thickening* OR xerification* and (2) soil* OR carbon* OR nutrient* OR heterogeneity*. A candidate database was then set up in which selection was performed according to the following rules: (1) the primary community of study areas is grasslands in which shrub-encroachment took place in the subsequent time period; (2) shrub encroachment was a natural process, not artificial plantation, and the study area has not been subjected to other experimental treatments such as N addition or precipitation control; (3) the encroaching species are mainly shrubs, and include some *Prosopis, Juiperus* and *Q*uercus species which with small body or shorter height, but do not include large body or taller trees and sub-shrubs; (4) the studies include pair-wise cases of both shrub-encroached sample areas and contrasting neighboring sample areas. In this study, we treated SEG as one vegetation type and compared it with neighboring non-SEG, with the non-SEG considered as the primary state before shrub encroachment occurred.

A total of 41 articles including 142 cases fit these criteria; most cases were located in arid and semi-arid areas, with only 13 cases in sub-humid and humid areas (see Fig. S1 and Table S1). The latitudes ranged from 43.10 °S to 52.15 °N and the longitudes from 112.12 °W to 48.13 °E; the average annual temperatures were between 6.9 and 28.4 °C, and the average annual precipitation was between 200 and 1065 mm.

Our datasets included soil organic carbon (SOC), longitude and latitude, mean annual precipitation (MAP), mean annual temperature (MAT), aridity index (AI), encroaching species, soil texture, soil bulk density (SBD), and soil total nitrogen (STN) content for each study site (both SEG and contrasting non-SEG sites). The aridity index (AI) was obtained from the Global Aridity Index (Global-Aridity) and the Global Potential Evapo-Transpiration (Global-PET) Geospatial Database (http://www.cgiar-csi.org/2010/04/134/); the other parameters were obtained from the literature.

### Data Analysis

The mean, standard deviation and sample size of the SOC of each pair-wise case were recorded. Given that deep soil and surface soil differ significantly in their response to shrub encroachment, this study focused on the soil within the depth of 0–50 cm and excluded those cases where soil depth exceeded 50 cm.

All data were classified into 4 climate zones based on the 1997 UNPE standard (Middleton & Thomas, 1997): arid, semi-arid, dry sub-humid, and humid. From our database, the soil samples were classified into 9 different soil textures within the USDA taxonomy system^27^,^28^: loam, silt, silt loam, clay loam, silty clay, silty clay loam, sand, sandy clay, and sandy clay loam. The soil depth was divided by 0–15 cm and 15–50 cm. To explore the impact of shrub species on SOC change, every shrub species was identified using extracted species information, and the “invasive” species were classified as legume and non-legume for analysis. There were 23 encroaching species belonging to 15 genera and 11 families, which included 10 species and 82 cases of legumes and 13 species and 55 cases of non-legumes.

The paired-plot method was used in the data source that we searched, which employs the effect size (log transformed response ratio)^29^ to explore the SOC differences between SEG and non-SEG^30^:


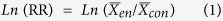


In equation (1) where RR is the response ratio, 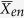 is the mean SOC content in SEG sample points, and 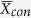 is the mean SOC content in control sample points. The variation of each response ratio is:


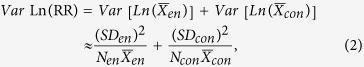


In equation (2) where *N*_en_ and *N*_*con*_ represent repeats of SOC measurements in shrub-encroached and contrast sample points, respectively. Likewise, *SD*_*en*_ and *SD*_*con*_ represent the standard deviations of SOC measurements in the shrub-encroached and contrast sample points, respectively. The effect size which quantifies the proportional difference between SOC content in SEG and non-SEG^30^. To avoid the small sample sizes bias, the standardized means (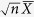/SD) for both mean values within each effect size should be generally >3 30. In our data set, only 2% of effect sizes fall below 3, while ~90% of scores exceed 6, suggest the log response ratio is appropriate.

The response ratio was calculated in the R package Metafor^31^. To account for the sampling dependence in our dataset, we used a hierarchical Bayes linear model (HBLM) in R package metahdep^32^. The HBLM is a method that allows controlling for sampling dependence^33^,^34^, something that is particularly important in our dataset which had multiple data points obtained within a given study (see Table S1). We analyzed 142 separate observations for subgroups within studies—that is, different climate class, shrub species and soil types and soil depth. A random effect was used to account for differences across studies, a grand mean effect size, across subgroups, was calculated using an intercept model^35^. The uncertainty in the regression coefficients is given by 95% credible intervals, two-sided *p* -values for the coefficients were also calculated for interpretation of significant effects. The potential for bias in published studies with larger sample sizes might have more power to detect significant impacts. The normal quartile plot (Fig. S2a) showed the studies were simulated to have a mean difference of 0.5 and a common variance of 1, indicating that the effect sizes are normally distributed however, the curve slightly skewed to the right and the long tail suggested some unpublished studies were deleted^36^. In combination with the funnel plot (Fig. S2b), we conducted a trim and fill assessment with 8 studies added, there was no significant impact to change the overall meaning of the results (0.0007 reduction in the effect size), so we are confident that our results were reliable^37^. The cumulative meta-analysis approach was used to assess publication bias and changes in the overall effect size along time (publication year) and soil depth (Fig. S3), which the grand mean effect size is robust over time and soil depth. As sample size added, the effect size tended to be stable and with narrower confidence interval, which increased the accuracy of our results. Moreover, we also done a regression analysis to explore the relationship between the average percentage change in SOC content and continuous variables such as average annual precipitation (MAP). For more details see Table S1.

We performed a simple structural equation modelling (SEM) to examine climate, species and soils on SOC change, due to the limit of dataset, we only used the category variable. However, we transformed the categorical variables of climate, soil texture and vegetation type into ordered variables, thus made the variable have relative amount^38^. Climate was ordered from arid to humid, soil texture was ordered by relative clay content from less to more, and non-legumes to legumes were ordered according to shrub type. In this frame work, climate and soils are supposed to influence species, while climate, soils and species all influence the SOC change. In the initial SEM model, we set climate and soil texture as exogenous variables, while shrub type and average percentage change in SOC content as the observed endogenous variables. e1 and e2, as two unobserved exogenous variables, represent the unexplained residuals in shrub type and SOC change. The SEM was conducted based on R package Lavaan^39^.

All statistical analysis were performed using the software package R 3.1.2 40.

## Results

SOC content tended to increase as shrubs encroached, with an effect value of 0.15 (*p* < 0.01, Table 1). The SOC increased more in soils (0–15 cm) than in deeper soils (15–5 cm) with an effect value of 0.20 vs. 0.073 (*p* < 0.01, Table 1), combined with the cumulative meta-analysis (Fig. S3), the soil depth not influence the total effect of shrub encroachment on SOC changes, so all the subsequent results were not make distinction of soil depth.

### SOC changes associated with climate

The SOC changes differed with climate zone: it increased significantly in semi-arid and humid areas, with effect values of 0.25 and 0.74, respectively (*p* < 0.01, Table 1), but did not change significantly in arid zones and dry sub-humid areas (*p* > 0.05, Table 1).

**Figure 1:**
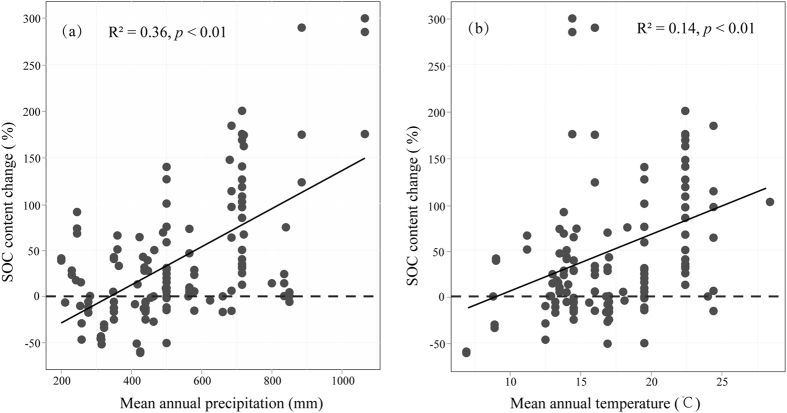
The relationships between the percentage of SOC content change (%) and the mean annual precipitation (MAP, **a**) and mean annual temperature (MAT, **b**).

A significant positive relationship was found between the average percentage change in SOC content and the MAP (R^2^ = 0.36, *p* < 0.01) and MAT (R^2^ = 0.14, *p* < 0.01). Shrub encroachment increased the SOC content in areas with relatively more precipitation and higher temperatures. The SOC increment was enhanced with the increase in temperature and annual precipitation, and the greatest SOC increment reached nearly 300% (Fig. 1).

### SOC changes with soil properties

SOC content increased significantly with shrub encroachment in sandy clay loam, sand, and sandy loam, with effect sizes of 0.61, 0.37, and 0.20, respectively (Table 1). In contrast, SOC significantly decreased with shrub encroachment in silty clay soil, with an effect size of −0.46 (*p* < 0.05), whereas shrub encroachment SOC was not significantly affected in other soil types (Table 1).

**Figure 2:**
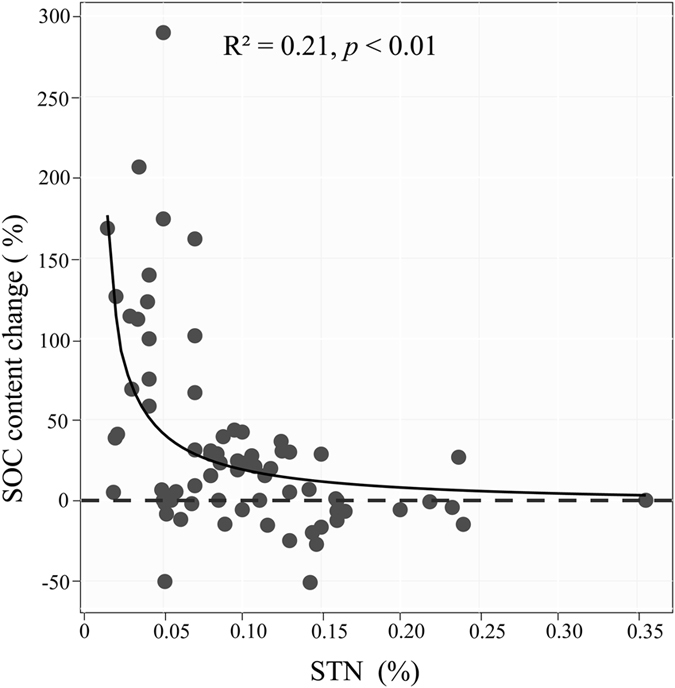
The relationship between the percentage of SOC content change (%) and the soil total nitrogen (STN) content (%).

**Figure 3:**
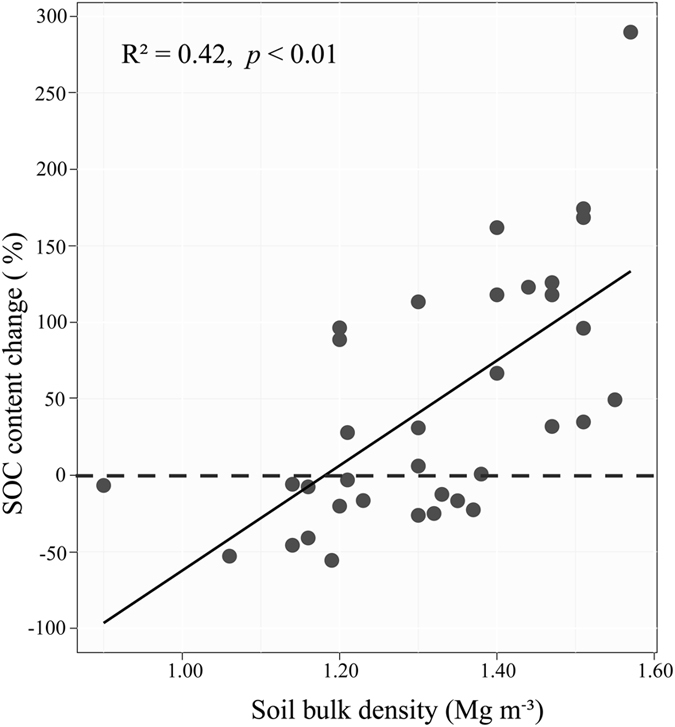
The relationship between the percentage of SOC content change (%) and the soil bulk density (Mg m^−^^3^).

Further analysis showed that the average percentage change in SOC content significantly decreased (a negative power relationship) with increasing soil nitrogen (STN) content (R^2^ = 0.21, *p* < 0.01, Fig. 2) but significantly increased with increasing soil bulk density (SBD) (R^2^ = 0.42, *p* < 0.01, Fig. 3).

### Effect of encroaching shrub species on SOC changes

The SOC content was significantly decreased with shrub encroachment induced by the genera *Acacia* and *Chuquiraga*, with effect values of −0.41 and −0.49, respectively (*p* < 0.01, Table 1). In contrast, the SOC content was significantly increased with encroachment induced by the genera *Juniperus, Myrica, Prosopis* and *Quercus*, with effect values of 0.34, 1.51, 0.61 and 0.19, respectively (*p* < 0.01, Table 1), and showed no significant change with the other encroaching shrub genera. Further analysis showed that encroachment by leguminous shrub species increased SOC more than did non-leguminous taxa, with an effect value of 0.32 vs. 0.15 (*p* < 0.01, Table 1).

### The determinants of S**O**C changes in relation to shrub encroachment

**Figure 4:**
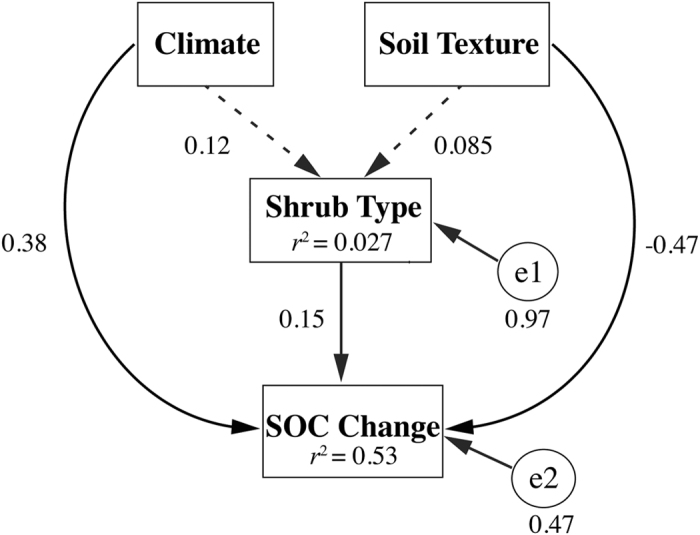
A structural equation model (SEM) showing the multivariate effects on SOC changes through hypothetical pathways of abiotic factors (climate and soil texture) and biotic factors (shrub types). The solid and dashed arrows indicate significant (p < 0.05) and non-significant (p > 0.05) effects, respectively; values associated with the arrows represent standardized path coefficients. R^2^ values associated with the response variables indicate the proportion of variation explained by relationships with other variables. The exogenous unobserved variables e1 and e2 account for the unexplained error in the estimation of shrub types and SOC change, respectively.

All the abiotic (climate and soil texture) and biotic (shrub type) variables had significant effects (p < 0.01) on SOC content changes, together accounting for 53% of the variance. The standardized direct path coefficients for climate, soil texture and shrub type to changes in SOC content were 0.38, 0.47 and 0.15, respectively, indicating that soil texture was the most important factor influencing the effect of shrub encroachment on SOC content (Fig. 4).

## Discussion

The SOC content could increase, decrease, or not change significantly in response to shrub encroachment^7^,^11^,^41^,^42^. Our study found that the shrub encroachment would increase SOC content in totally, however, the changes direction and magnitude of SOC content with shrub encroachment varied with abiotic (climate and soil) and biotic (plant) factors.

Our results suggested that shrub encroachment decreased SOC content in the areas with less precipitation and lower temperatures and increased SOC content in areas with relatively abundant precipitation and higher temperatures. The SOC content gain or loss with shrub encroachment is mainly driven by the relative difference in productivity between SEG and non-SEG^43^. Studies have demonstrated that increased precipitation significantly increases grassland ANPP and promotes SOC accumulation in northern China (Bai *et al*. 2010; Liu *et al*. 2009). In contrast, increased temperature can have variable impacts on soil C storage; depending on precipitation conditions, soil properties and other factors, high temperatures can cause warming-induced water stress or inhibit photosynthesis in the herbaceous layer, resulting in a reduced grassland ANPP and SOC storage. However, in SEG, under abundant precipitation and high temperature situations, the ANPP increased^8^,^43^, due to shrubs’ having greater tolerance of high temperatures, and shaded herbs from temperature stress, which favor maintaining high photosynthetic rates^44^. Thus, the different adaptive strategies to temperature and water conditions employed by grass and shrub species likely contribute substantially to the variation in SOC accumulation in response to shrub encroachment, though field data on this phenomenon are limited. Studies have shown shrublands contribute a large proportion of recalcitrant or long-lived carbon, yet, grasslands have high concentrations of labile carbon^45^. Thereby higher growth of shrub in higher precipitation and temperature environment made SEGs accumulate more SOC than control grasslands.

Our results indicated that shrub encroachment in dry sub-humid and humid zones did not have a significant or positive effect on SOC content, differing from previous studies, which found that the SOC content decreased or experienced no changes in response to shrub encroachment in humid areas^7^,^46^,^47^. This was partly because little studies have conducted, Such as Jackson *et al*.^7^ only had 2 site in humid zone, and in this paper only 3 studies in sub-humid areas, thus, it is difficult to fit the general climate gradient. More importantly, the climate zones were not only the combination of temperature and precipitation in a given region, but also influenced by other factors, such as sunshine duration, soil properties, topography, relative evaporation and so on, which could also directly or indirectly affect SOC dynamics. Through carefully examining the dataset, we found those cases we mentioned above were all conducted in the areas with greater silt and clay content in soils. However, the maximum increment of SOC content (nearly 300%) from our dataset was conducted by Brantley, *et al*. in humid sites with higher sand content^41^.

Taken together, these comparisons suggest that climate alone cannot explain the variation in SOC changes in relation to shrub encroachment and its geographical gradient. Combine with the results from our SEM model, which the soil texture had the greatest explanatory capacity for the change in SOC content, we proposed that the soil properties were more important for the SOC dynamics accounted for the shrub encroachment. Soil texture determines the rate of SOC turnover^48^,^49^, and fine particle soils could form stable organomineral complexes with physical protection, prevent SOC decomposition^50^. However, the potential for shrub encroachment continue to accumulate SOC may be different, some studies have clearly shown that soil texture plays negative role in the change of soil content after shrub encroached into grasslands. From one aspect, relative dominance of shrubs in grassland was found to correlate with soil texture^51^, the shrubs made up a larger proportion of total ANPP at the coarse textured sites than fine textured sites with the same shrub cover^52^, indicating when shrub encroached into grasslands at coarse textured sits, shrub would contribute to more biomass carbon for SOC accumulation. From other aspect, coarse textured soils tend to have higher bulk density^53^, we found a significant positive relationship exists between the average percentage change in SOC content and soil bulk density, in line with the results of soil texture. In accordance to the former studies, with the encroachment of C3 shrub into C4 plant dominated grasslands, the accumulation of SOC in sandy loam soil was approximately 23% higher than in clay loam soil under the same precipitation and temperature conditions^54^,^55^. Evidencing from carbon isotope δ^13^ C analysis that C3 plant derived carbon is preferentially added into the coarse textured soils than in fine textured soil, while the C4 plant derived carbon just the other way^56^.

The results from the STN analysis in our study (Fig. 2) further demonstrated the importance of soil properties for the effect of shrub encroachment on SOC content, regarding the control of SOC by soil N content^57^. Based on a global meta-analysis, an increase of 1 g N would promote the accumulation of 7–13 g C^58^. Shrub encroachment reportedly promotes the accumulation of soil N^17,41^ and thus has an accumulation effect on the SOC content. However, with increased STN, increment of SOC content is reduced and tends to remain constant. One potential explanation for this result is shrub encroachment resulting in unbalanced carbon and nitrogen budgets, and the ability of soils to preserve SOC have a maximum concentration that is referred to as the soil protective capacity^59^. It was reported that the C: N ratio is larger in coarse textured soils than fine texture^60^, this suggested the coarse textured soils with low STN content have higher capacity to accumulate SOC. Modeling the effect of shrub encroachment on SOC change only with soil texture difference, showed the similar results with us^21^.

Our current study also confirms that different shrub species affect SOC accumulation differently (Table 1). Previous studies showed that different shrub species have different impacts on the activity of soil microorganisms, ANPP and root biomass^61^, which contributes to SOC accumulation and influence the protection of SOC against decomposition. For example, N-fixing species such as *Dichrostachys cinerea* have the potential to overcome particular nutrient constraints to promote carbon accumulation^62^, compared with other species, the N-fixing could accumulate 30% more SOC, according to a meta-analysis conducted by Johnson & Curtis^63^.

Other factors may also influence the effect of shrub encroachment on SOC, as show in SEM model there still has 47% uncertainty cannot be explained in this study which should be further identified. The time of shrub encroachment, shrub density and human activity, such as grazing intensity and other activities, can all play a significant role depending on other conditions. Information on the specific duration of encroachment and other conditions noted above were not examined in this study. However, the cumulative meta-analysis reveals that the grand mean effect is highly relevant to time (Fig. S3a), and interestingly, we found many of the earliest studies in our dataset reported that the shrub encroachment would decrease SOC, this in some ways suggest the effect of shrub encroachment on SOC was closely related to encroached time or age, a recent research demonstrates changes in SOC with 34 years *Caragana microphylla* plantation was increased after decreased at first^64^. Besides, we only focused on the soil within the depth range of 0–50 cm, with the increase of soil depth, has been not shown to affect our total result (Fig. S3b), and the 0–15 cm soil show more SOC change than 15–30 cm soils, but the impact of shrub encroachment on more deep-layer SOC may be different, considering the fact shrub have a deeper and different root system compared with herbs^7^, we expecting improvements in the following studies. To obtain a better understanding of shrub encroachment, more factors must be carefully examined. In addition, the datasets in this paper contain limited information from Asia, where shrub encroachment covers large areas. Thus, we recommend additional studies investigating Asian areas in future.

## Conclusions

This study demonstrates the mainly positive effect of shrub encroachment on SOC content. However, this effect varies among climate, soil and shrub types. We highlight the role of antecedent soil properties, which determined the changeability of SOC content caused by shrub encroachment: coarse textured soils have a greater capacity to increase the SOC content, and the increment can be enhanced by higher precipitation and temperature as well as by encroachment by leguminous shrubs rather than by non-leguminous shrubs. Our study provides insight for the further understanding of land cover transformation as well as for the conservation and management of grasslands.

## Additional Information

**How to cite this article**: Li, H. *et al*. Effects of shrub encroachment on soil organic carbon in global grasslands. *Sci. Rep.* **6**, 28974; doi: 10.1038/srep28974 (2016).

## Supplementary Material

Supplementary Information

Supplementary Table S1

## Figures and Tables

**Table 1:** Estimates of the effect size (log response ratios) of SOC content influenced by shrub encroachment for different variable classes across climate class, shrub type, soil texture and soil depth with 95% confidence intervals (CI).

Variable class	*n*	Effect size	CI	*p*
**Climate class**				
Arid	20	0.029	−0.048~0.11	0.45
Semi-arid	109	0.16	0.15~0.34	<0.0001
Dry sub-humid	3	−0.0062	−0.18~0.15	0.94
Humid	11	0.45	0.34~0.54	<0.0001
**Shrub types**				
**Genus level**				
*Acacia*	6	−0.40	−0.69~−0.14	<0.0001
*Brachystegia*	3	1.09	0.67~1.46	<0.0001
*Callitris*	7	−0.024	−0.27~0.24	0.67
*Caragana*	5	-0.26	−0.54~0.06	0.0011
*Chuquiraga*	2	−0.49	−0.95~−0.02	<0.0001
*Cornus*	1	0.0028	−0.66~0.66	0.98
*Eucalyptus*	1	−0.045	−0.71~0.62	0.72
*Juniperus*	9	0.16	0.11~0.58	0.00081
*Larrea*	18	0.059	−0.15~0.22	0.12
*Mulinum*	1	−0.11	−0.78~0.56	0.43
*Myrica*	3	1.22	1.16~1.86	<0.0001
*Prosopis*	33	0.61	0.49~0.73	<0.0001
*Quercus*	37	0.037	0.06~0.31	0.23
*Rosmarinus*	5	0.21	−0.08~0.53	0.035
*Symphoricarpos*	11	−0.12	−0.32~0.10	0.024
**Family level**				
Leguminosae	84	0.21	0.17~0.27	<0.0001
Non-Leguminosae	58	0.081	0.033~0.13	0.0008
**Soil texture**				
Clay loam	3	−0.045	−0.29~0.21	0.72
Loam	33	0.075	−0.03~0.15	0.058
Sand	4	0.42	0.27~0.58	<0.0001
Sandy clay loam	25	0.59	0.50~0.67	<0.0001
Silt	5	0.034	−0.095~0.16	0.61
Silty clay	4	−0.44	−0.59~−0.30	<0.0001
Silty clay loam	3	0.13	−0.18~0.44	0.40
Silty loam	4	0.22	−0.042~0.47	0.098
Sandy loam	36	0.19	0.13~0.24	<0.0001
**Soil depth**				
0–15 cm	76	0.20	0.15~0.24	0.0023
15–30 cm	66	0.073	0.026~0.16	<0.0001
**Overall**	142	0.15	0.11~0.18	<0.0001

The number of observations (*n*) and significance levels (*p*) are reported for each variable.
