# Supplementary material for: Effects of shrub encroachment on soil organic carbon in global grasslands
Source: Sci Rep. 2016 Jul 8;6:28974. doi: 10.1038/srep28974 (PMC4937411; doi:10.1038/srep28974)
Supplement: Supplementary Information [file srep28974-s1.docx]

**Effects of shrub encroachment on soil organic carbon in global grasslands**

**He Li, Haihua Shen, Leiyi Chen, Taoyu Liu, Huifeng Hu, Xia Zhao, Luhong Zhou, Pujin Zhang, Jingyun Fang^*^**

1. State Key Laboratory of Vegetation and Environmental Change, Institute of Botany, Chinese Academy of Sciences, Beijing 100093, China

2. University of the Chinese Academy of Sciences, Beijing, 100049, China

3. Department of Ecology, College of Urban and Environment, and Key Laboratory of Earth Surface Processes of the Ministry of Education, Peking University, Beijing 100871, China

4. Inner Mongolia Prataculture Research Center, Chinese Academy of Sciences, Hohhot010031, China

***Corresponding authors**

Jingyun Fang, Professor

Department of Ecology, Peking University

Beijing 100871, China

Tel/Fax: +86 10 6275 6560;

E-mail: [jyfang@urban.pku.edu.cn](mailto:jyfang@urban.pku.edu.cn)

**Running Title: Shrub encroachment on soil organic carbon**

This supplementary information file contains Table S1, Figure S1-3, and references.

**Supplementary information**

**Table S1** (xls format)**:** Information on study sites used in this study, including location, climate zone, mean annual temperature (MAT), mean annual precipitation (MAP), shrub species, response ratio and others. See separate Excel sheet.

**Figure S1.** Global distribution of shrub encroachment studies included in this meta-analysis. The maps have been produced using Arcgis 10.0, background imag was the map of Global Aridity Index which provided online (http://www.cgiar-csi.org)by the CGIAR-CSI with the support of the International Center for Tropical Agriculture (CIAT).


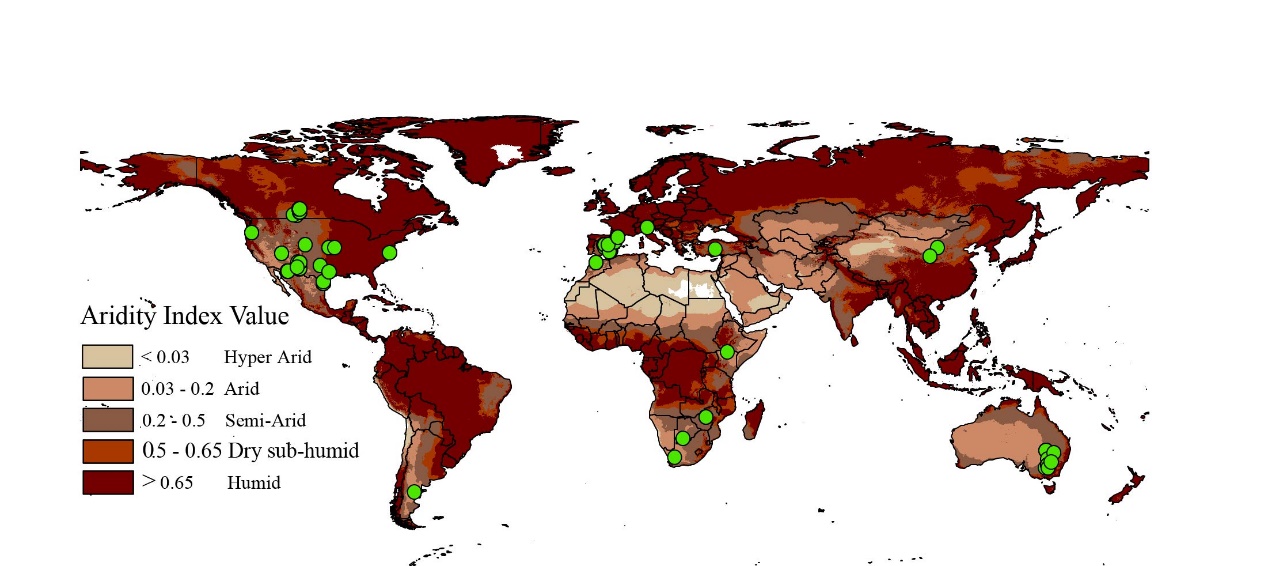


**Figure S2.** (a) Normal quartile plot for simulated data set with a mean of 0.5 and standard deviation of 1 (N = 142), (b) Funnel plot of residuals versus inverse of standard error. The red dashed line shows 95% confidence limits. The maps have been produced using R 3.2.


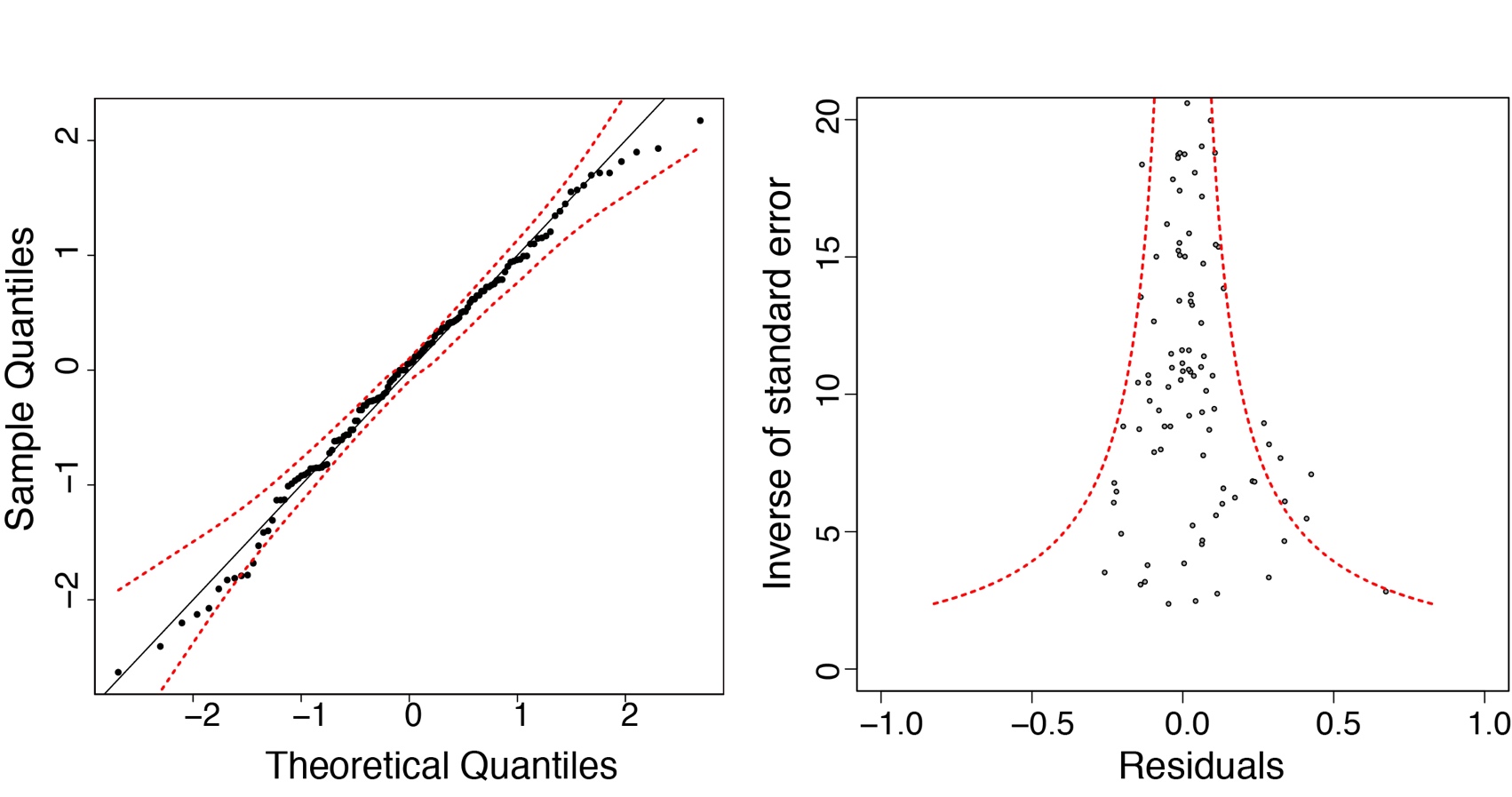


（a）

（b）

**Figure S3.** Cumulative meta-analysis forest plot of data sorted by increasing (a) publication date and (b) soil depth. The maps have been produced using R 3.2.


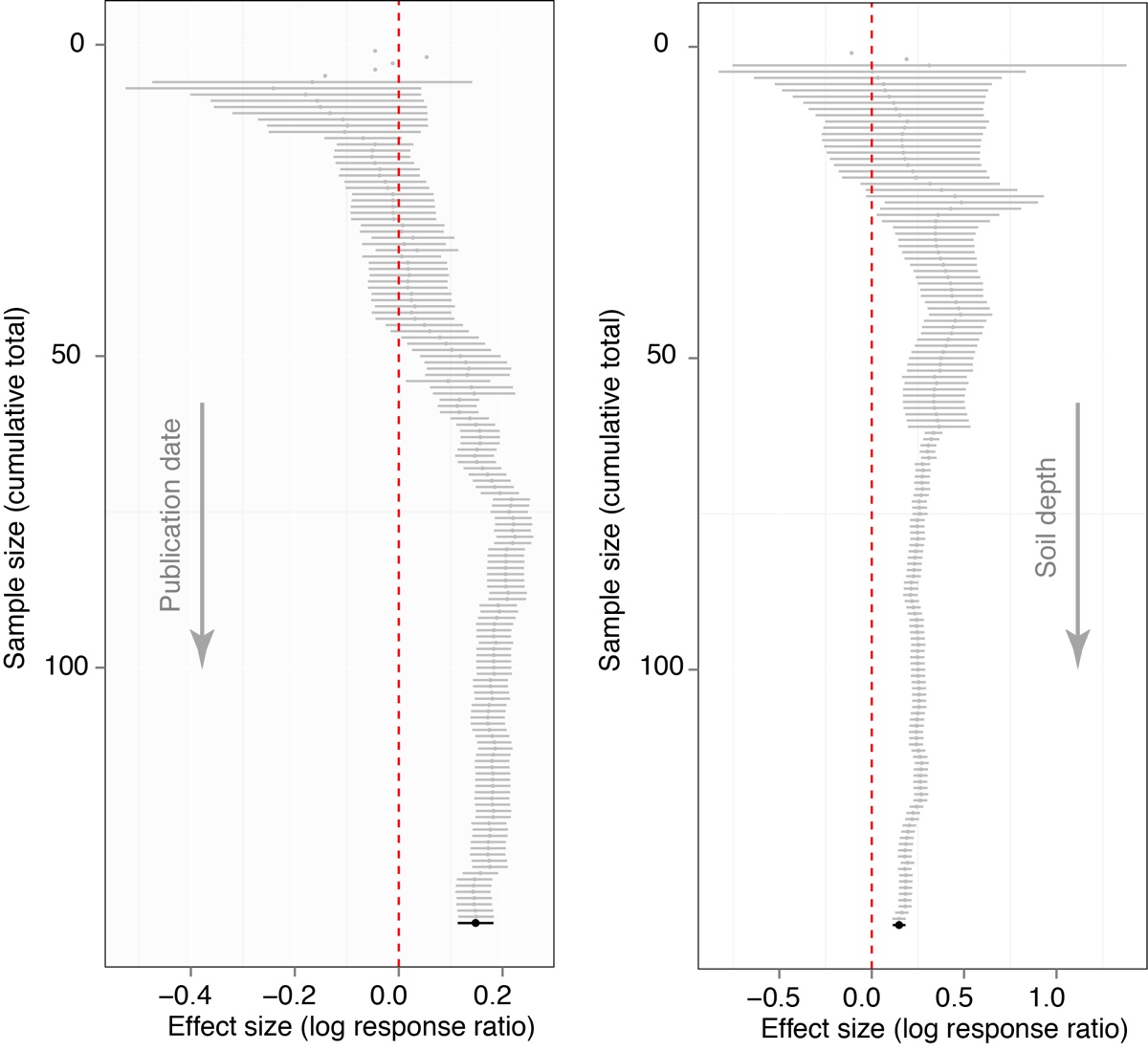


（a）

（b）

**Reference**[^1-41^](#_ENREF_1)

1. Angassa, A., Sheleme, B., Oba, G., Treydte, A. C., Linstadter, A. & Sauerborn, J. Savanna land use and its effect on soil characteristics in southern Ethiopia. *J. Arid Environ.* **81**, 67-76 (2012).

2. Bai, Y., Colberg, T., Romo, J., McConkey, B., Pennock, D. & Farrell, R. Does expansion of western snowberry enhance ecosystem carbon sequestration and storage in Canadian Prairies? *Agr. Ecosyst. Environ.* **134**, 269-276 (2009).

3. Boutton, T. W., Archer, S. R., Midwood, A. J., Zitzer, S. F. & Bol, R. [delta] 13C values of soil organic carbon and their use in documenting vegetation change in a subtropical savanna ecosystem. *Geoderma* **82**, 5-41 (1998).

4. Brantley, S. T. & Young, D. R. Shrub expansion stimulates soil C and N storage along a coastal soil chronosequence. *Glob. Chang Biol.* **16**, 2052-2061 (2010).

5. Brazier, R. E., Turnbull, L., Wainwright, J. & Bol, R. Carbon loss by water erosion in drylands: implications from a study of vegetation change in the south-west USA. *Hydrol. Process.* **28**, 2212-2222 (2014).

6. Briggs, J. M., Knapp, A. K., Blair, J. M., Heisler, J. L., Hoch, G. A., Lett, M. S. & McCarron, J. K. An ecosystem in transition. Causes and consequences of the conversion of mesic grassland to shrubland. *Bioscience* **55**, 243-254 (2005).

7. Chartier, M. P., Rostagno, C. M. & Videla, L. S. Selective erosion of clay, organic carbon and total nitrogen in grazed semiarid rangelands of northeastern Patagonia, Argentina. *J. Arid Environ.* **88**, 43-49 (2013).

8. Colberg, T. J. *Relationships between plant communities and soil carbon in the prairie ecozone of Saskatchewan*, UNIVERSITY OF SASKATCHEWAN, (2007).

9. Connin, S. L., Virginia, R. A. & Chamberlain, C. P. Carbon isotopes reveal soil organic matter dynamics following arid land shrub expansion. *Oecologia* **110**, 374-386 (1997).

10. Daryanto, S., Eldridge, D. J. & Throop, H. L. Managing semi-arid woodlands for carbon storage: Grazing and shrub effects on above- and belowground carbon. *Agr. Ecosyst. Environ.* **169**, 1-11 (2013).

11. Evrendilek, F., Celik, I. & Kilic, S. Changes in soil organic carbon and other physical soil properties along adjacent Mediterranean forest, grassland, and cropland ecosystems in Turkey. *J. Arid Environ.* **59**, 743-752 (2004).

12. Gonzalez-Roglich, M., Swenson, J. J., Jobbagy, E. G. & Jackson, R. B. Shifting carbon pools along a plant cover gradient in woody encroached savannas of central Argentina. *Forest Ecol. Manag.* **331**, 71-78 (2014).

13. Hagos, M. G. & Smit, G. N. Soil enrichment by Acacia mellifera subsp detinens on nutrient poor sandy soil in a semi-arid southern African savanna. *J. Arid Environ.* **61**, 47-59 (2005).

14. Hibbard, K. A. *Landscape patterns of carbon and nitrogen dynamics in a subtropical savanna: observations and models*, Texas A & M University, (1995).

15. Hibbard, K. A., Archer, S., Schimel, D. S. & Valentine, D. W. Biogeochemical changes accompanying woody plant encroachment in a subtropical savanna. *Ecology* **82**, 1999-2011 (2001).

16. Jackson, R. B., Banner, J. L., Jobbagy, E. G., Pockman, W. T. & Wall, D. H. Ecosystem carbon loss with woody plant invasion of grasslands. *Nature* **418**, 623-626 (2002).

17. Kieft, T. L., White, C. S., Loftin, S. R., Aguilar, R., Craig, J. A. & Skaar, D. A. Temporal dynamics in soil carbon and nitrogen resources at a grassland-shrubland ecotone. *Ecology* **79**, 671-683 (1998).

18. King, J. A. & Campbell, B. M. Soil organic matter relations in five land cover types in the miombo region (Zimbabwe). *Forest Ecol. Manag.* **67**, 225-239 (1994).

19. Koteen, L. E., Raz-Yaseef, N. & Baldocchi, D. D. Spatial heterogeneity of fine root biomass and soil carbon in a California oak savanna illuminates plant functional strategy across periods of high and low resource supply. *Ecohydrology* **8**, 294-308 (2015).

20. Lett, M. S., Knapp, A. K., Briggs, J. M. & Blair, J. M. Influence of shrub encroachment on aboveground net primary productivity and carbon and nitrogen pools in a mesic grassland. *Can. J. Bot.* **82**, 1363-1370 (2004).

21. Liao, J. D. & Boutton, T. W. Soil microbial biomass response to woody plant invasion of grassland. *Soil Biol. Biochem.* **40**, 1207-1216 (2008).

22. Liu, F., Archer, S. R., Gelwick, F., Bai, E., Boutton, T. W. & Wu, X. B. Woody plant encroachment into grasslands: spatial patterns of functional group distribution and community development. *PLoS One* **8**, e84364 (2013).

23. Maestre, F. T., Bowker, M. A., Puche, M. D., Hinojosa, M. B., Martinez, I., Garcia-Palacios, P., Castillo, A. P., Soliveres, S., Luzuriaga, A. L., Sanchez, A. M., Carreira, J. A., Gallardo, A. & Escudero, A. Shrub encroachment can reverse desertification in semi-arid Mediterranean grasslands. *Ecol. Lett.* **12**, 930-941 (2009).

24. Martí-Roura, M., Casals, P. & Romanyà, J. Temporal changes in soil organic C under Mediterranean shrublands and grasslands: impact of fire and drought. *Plant Soil* **338**, 289-300 (2010).

25. McCulley, R. L., Archer, S. R., Boutton, T. W., Hons, F. M. & Zuberer, D. A. Soil respiration and nutrient cycling in wooded communities developing in grassland. *Ecology* **85**, 2804-2817 (2004).

26. McKinley, D. C. & Blair, J. M. Woody plant encroachment by Juniperus virginiana in a mesic native grassland promotes rapid carbon and nitrogen accrual. *Ecosystems* **11**, 454-468 (2008).

27. Mcpherson, G. R., Boutton, T. W. & Midwood, A. J. Stable Carbon Isotope Analysis of Soil Organic-Matter Illustrates Vegetation Change at the Grassland Woodland Boundary in Southeastern Arizona, USA. *Oecologia* **93**, 95-101 (1993).

28. Moreno, G. & Obrador, J. J. Effects of trees and understorey management on soil fertility and nutritional status of holm oaks in Spanish dehesas. *Nutri. Cycl. Agroecosyst.* **78**, 253-264 (2007).

29. Neff, J. C., Barger, N. N., Baisden, W. T., Fernandez, D. P. & Asner, G. P. Soil carbon storage responses to expanding pinyon-juniper populations in southern Utah. *Ecol. Appl.* **19**, 1405-1416 (2009).

30. Oliver, I. A. The pilliga biodiversity dataset. Unpublished data,Department of Environment, Climate Change and Water, Armidale.

31. Parizek, B., Rostagno, C. M. & Sottini, R. Soil erosion as affected by shrub encroachment in northeastern Patagonia. *J. Range Manage.* **55**, 43-48 (2002).

32. Pei, S. F., Fu, H. & Wan, C. G. Changes in soil properties and vegetation following exclosure and grazing in degraded Alxa desert steppe of Inner Mongolia, China. *Agr. Ecosyst. Environ.* **124**, 33-39 (2008).

33. Puttock, A., Dungait, J. A. J., Macleod, C. J. A., Bol, R. & Brazier, R. E. Woody plant encroachment into grasslands leads to accelerated erosion of previously stable organic carbon from dryland soils. *J. Geophys. Res.: Biogeosci.* **119**, 2345-2357 (2014).

34. Qiu, L. P., Wei, X. R., Zhang, X. C., Cheng, J. M., Gale, W., Guo, C. & Long, T. Soil organic carbon losses due to land use change in a semiarid grassland. *Plant Soil* **355**, 299-309 (2012).

35. Quero, J. L., Maestre, F. T., Ochoa, V., Garcia-Gomez, M. & Delgado-Baquerizo, M. On the Importance of Shrub Encroachment by Sprouters, Climate, Species Richness and Anthropic Factors for Ecosystem Multifunctionality in Semi-arid Mediterranean Ecosystems. *Ecosystems* **16**, 1248-1261 (2013).

36. Smith, D. L. & Johnson, L. C. Expansion of Juniperus virginiana L. in the Great Plains: Changes in soil organic carbon dynamics. *Glob. Biogeochem. Cycles* **17**, 1062 (2003).

37. Thompson, W. A. & Eldridge, D. J. Plant cover and composition in relation to density of Callitris glaucophylla (white cypress pine) along a rainfall gradient in eastern Australia. *Aust. J. Bot.* **53**, 545-554 (2005).

38. Throop, H. L., Lajtha, K. & Kramer, M. Density fractionation and 13C reveal changes in soil carbon following woody encroachment in a desert ecosystem. *Biogeochemistry* **112**, 409-422 (2013).

39. Turnbull, L., Wainwright, J., Brazier, R. E. & Bol, R. Biotic and Abiotic Changes in Ecosystem Structure over a Shrub-Encroachment Gradient in the Southwestern USA. *Ecosystems* **13**, 1239-1255 (2010).

40. Virginia, R. A., Jarrell, W. M., Whitford, W. G. & Freckman, D. W. Soil Biota and Soil Properties in the Surface Rooting Zone of Mesquite (Prosopis-Glandulosa) in Historical and Recently Desertified Chihuahuan Desert Habitats. *Biol. Fertil. Soils* **14**, 90-98 (1992).

41. Wang, X. P., Brown-Mitic, C. M., Kang, E. S., Zhang, J. G. & Li, X. R. Evapotranspiration of Caragana korshinskii communities in a revegetated desert area: Tengger Desert, China. *Hydrol. Process.* **18**, 3293-3303 (2004).
